# Successful reuse of a transplanted kidney 9 years after initial transplantation: 4-year follow-up

**DOI:** 10.1186/s12882-018-1040-0

**Published:** 2018-09-17

**Authors:** Wen-Hsin Tseng, Yu-Feng Tian, Alex Chien-Hwa Liao, Ming-Jenn Chen, Hsuan-Ying Ho, Jinn-Rung Kuo, Steven K. Huang

**Affiliations:** 1 Division of Urology, Department of Surgery, Chi Mei Medical Center, Tainan, Taiwan; 2 Division of Transplantation Surgery, Division of General Surgery, Department of Surgery, Chi Mei Medical Center, Tainan, Taiwan; 3 Department of Health and Nutrition, Chia Nan University of Pharmacy and Science, Tainan, Taiwan; 4 Division of Urology, Division of Transplantation Surgery, Department of Surgery, Chi Mei Medical Center, Tainan, Taiwan; 5 Division of Neurosurgery, Department of Surgery, Chi Mei Medical Center, Tainan, Taiwan; 6 Department of Biotechnology, Southern Taiwan University of Science and Technology, Tainan, Taiwan

**Keywords:** Kidney transplantation, Reuse

## Abstract

**Background:**

Kidney transplantation is the preferred renal replacement therapy for patients with end-stage renal disease, but the waiting list for kidneys continues to grow because of a shortage of donor organs. The reuse of transplanted kidneys would seem to be a good approach to expand the pool of available organs. Here, we describe the reuse of a kidney 9 years after the initial transplantation. At 4-year follow-up, the second recipient is showing good renal function.

**Case presentation:**

In 2005, a kidney was transplanted from a 40-year-old man, who suffered brain death due to an intracranial hemorrhage, into a 45-year-old man. Nine years later, the recipient suffered a ruptured cerebral aneurysm, resulting in brain death. The kidney was re-transplanted into a 40-year-old man with diabetic nephropathy who had received hemodialysis for 5 years. During 4 years of follow-up, the graft has functioned well.

**Conclusions:**

This case demonstrates the successful regrafting of a transplanted kidney. We believe this is the longest period for reuse of kidney after initial transplantation. The outcome suggests that a well-functioning transplanted kidney can be reused years after transplantation.

## Background

Despite the transplantation of kidneys from expanded criteria donors [1], the waiting list for kidneys continues to grow because of a shortage of donor organs [2, 3]. In 1987, Al-Hasani et al. first reported the reuse of a transplanted kidney [4]. This seemed to offer a good approach for expanding the pool of available organs; however, the reuse of a transplanted kidney has rarely been attempted, with only around 10 cases reported [4–13]. Here, we describe a case in which a kidney was reused 9 years after the initial transplantation, probably the longest period after the initial transplantation for the reuse of a kidney. After 4 years of follow-up, the second recipient continues to show good renal function.

## Case presentation

The first donor was a 40-year-old man who suffered brain death due to intracranial hemorrhage after a traffic accident. His terminal serum creatinine level was 0.8 mg/dL and his Kidney Donor Profile Index score was 27%. The first recipient was a 45-year-old man with a 20-year history of hypertension and end-stage renal disease (ESRD) due to hypertensive nephropathy, who had received regular hemodialysis for 2 years. In June 2005, at another hospital, single renal transplantation was performed in the right iliac fossa, with a cold ischemia time of 5 h 10 min and a warm ischemia time of 1 h 48 min. After reperfusion, the recipient immediately passed urine. He was administered an immunosuppressive regimen comprising methylprednisolone, cyclosporine, everolimus, and mycophenolate mofetil, and he was discharged 10 days after the transplantation with a serum creatinine level of 1.4 mg/dL. At regular follow-up over the next 6 months, his serum creatinine levels remained within the normal range.

**Fig. 1:**
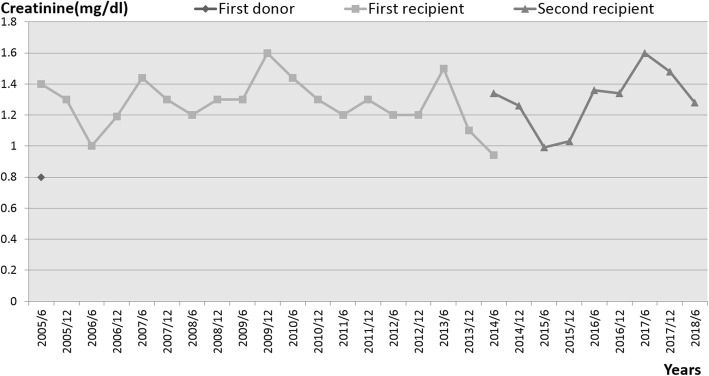
Creatinine levels of the first donor and the two recipients, measured every 6 months for 13 years

Over the following 9 years, the recipient showed no episodes of rejection, and his serum creatinine levels and creatinine clearance rates were within the normal ranges (Fig. 1). In 2010, he underwent coronary percutaneous angioplasty and stent placement for coronary artery disease, and thereafter he regularly took aspirin. However, in June 2014, he suffered a right cerebral aneurysm rupture that resulted in brain death. At that time, his serum creatinine level was 0.94 mg/dL and the creatinine clearance rate was 90 mL/min. Before his death, the patient (while completely conscious) and his family had expressed a wish for his organs to be donated; we therefore harvested the transplanted kidney for reuse.

The second recipient of the kidney was a 40-year-old man with ESRD caused by diabetic nephropathy, who had been undergoing hemodialysis for 5 years and had been added to the waiting list for renal transplantation at that time. His blood group was the same as that of the initial donor and the first recipient (A rhesus positive). There were four human leukocyte antigen mismatches with the original donor and two with the second donor (Table 1). Crossmatching with the initial donor was not possible because of the long time that had elapsed since the initial transplantation, but crossmatching with the second donor was negative. A biopsy demonstrated the quality of the donated kidneys: the Remuzzi score was 1 and the Kidney Donor Profile Index score was 74%.

**Table 1:** Characteristics of the first donor and the two recipients

	First Donor	First recipient/ Second donor	Second recipient
Sex	Male	Male	Male
Age (years)			
Transplantation:		45	40
Death:	40	54	
Cause of death	Intracranial hemorrhage	Ruptured cerebral aneurysm	
Blood group	A positive	A positive	A positive
Human leukocyte antigen	A03	A11	A11
	A24	–	A31
	B35	B46	B46
	B46	B75	–
	DR01	DR09	DR08

Kidney transplantation was performed in June 2014, with a cold ischemia time of 4 h 12 min and a warm ischemia time of 1 h 12 min. After transplantation, the recipient was administered an induction immunosuppressive regimen comprising basiliximab, high-dose methylprednisolone, and cyclosporine, subsequently shifted gradually to a maintenance immunosuppressive regimen comprising prednisolone, tacrolimus, everolimus, and mycophenolate mofetil. The second recipient was discharged 13 days after the transplantation, and his serum creatinine level was measured at follow-up every 3 months. As of June 2018, his renal function has remained stable, with a serum creatinine level of around 1.24 mg/dL (Fig. 1). There have been no episodes of rejection, and the patient has remained in a good clinical condition.

## Discussion and conclusion

Kidney transplantation is an effective approach for patients with ESRD but is limited by the shortage of kidney donations. To address this, in 2003, the Board of Directors of the Organ Procurement and Transplantation Network/United Network for Organ Sharing adopted a new expanded criteria donor allocation policy for the USA, which defined an expanded criteria donor as any brain-dead donor aged > 60 years or a donor aged > 50 years with any two of the following three conditions: a history of hypertension, a terminal serum creatinine level ≥ 1.5 mg/dL, or death resulting from a cerebrovascular accident [1]. Studies have reported that patients who received a kidney from an expanded criteria donor experienced better outcomes than those who remained on dialysis therapy [14].

The most common cause of the loss of a kidney allograft is the death of a patient with a functioning graft [11, 15, 16]. In the 30 years since Al-Hasani et al. first reported the reuse of a transplanted kidney [4], there have been only 10 case reports of the reuse of a kidney [4–13]. Of the 10 initial recipients of these kidneys, six suffered brain death due to a massive intracranial hemorrhage of unknown cause within 14 days after transplantation. Our case, with the first recipient experiencing 9 years of good renal function, is likely to have involved the longest period before the reuse of a kidney.

There has been speculation regarding the potential additive effects of repeated ischemia/reperfusion injury and the immunologic host response [5, 7]. Kidney grafts have been reused less frequently than liver grafts; this is mainly because liver transplantation is a lifesaving procedure for patients with end-stage liver disease, whereas some patients with ESRD have to remain on hemodialysis [13]. In the present case, however, the second recipient showed good renal and urinary functions immediately after the transplantation, and the graft has functioned well for 4 years.

The long-term outcome of kidney re-transplantation depends on several variables, including the kidney’s exposure to repeated ischemia/reperfusion and brain stem death in two patients [5, 7]. The outcome is likely to be less favorable than that for a kidney that is not being reused, which calls into question the appropriateness of offering such an organ for re-transplantation, as well as raising a number of ethical and scientific dilemmas [7]. Based on previous experience, we ensured the second recipient in this case was completely aware of the source of the kidney and the increased risk to which he would be exposed. As with other patients on the waiting list, our patient was willing to accept the risk of the surgery because he had been waiting for this opportunity for 5 years. Furthermore, the second donor and his family members seemed to have a strong understanding of the importance of organ donation, making them more prone to supporting the donation even of a previously transplanted organ. These observations may provide evidence for the ethical good of reusing organs for transplantation.

It is rare for a transplanted kidney to be reused, but such reuse may offer a viable option for increasing the pool of available organs. In the present case, a transplanted kidney was used 9 years after the initial transplantation; to the best of our knowledge, this is the longest period after the initial transplantation for the reuse of a kidney. The patient’s renal function has remained good throughout 4 years of follow-up. Our results suggest that a transplanted kidney can be re-harvested if the second donor’s renal function is normal.

## Abbreviation

ESRD: End-stage renal disease
